# Multiple *FLC* haplotypes defined by independent *cis*-regulatory variation underpin life history diversity in *Arabidopsis thaliana*

**DOI:** 10.1101/gad.245993.114

**Published:** 2014-08-01

**Authors:** Peijin Li, Daniele Filiault, Mathew S. Box, Envel Kerdaffrec, Cock van Oosterhout, Amity M. Wilczek, Johanna Schmitt, Mark McMullan, Joy Bergelson, Magnus Nordborg, Caroline Dean

**Affiliations:** 1 John Innes Centre, Norwich NR4 7UH, United Kingdom;; 2 Gregor Mendel Institute, Austrian Academy of Sciences, 1030 Vienna, Austria;; 3 Department of Environmental Sciences, University of East Anglia, Norwich NR4 7TJ, United Kingdom;; 4 Deep Spring College, Big Pine, California 93513, USA;; 5 University of California at Davis, Davis, California 95616, USA;; 6 Department of Ecology and Evolution, University of Chicago, Chicago, Illinois 60637, USA

**Keywords:** allelic heterogeneity, *FLOWERING LOCUS C*, noncoding polymorphism, vernalization, adaptation

## Abstract

Relating molecular variation to phenotypic diversity is a central goal in evolutionary biology. In *Arabidopsis thaliana*, *FLOWERING LOCUS C* (*FLC*) is a major determinant of variation in vernalization—the acceleration of flowering by prolonged cold. Here, through analysis of 1307 *A. thaliana* accessions, we identify five predominant *FLC* haplotypes defined by noncoding sequence variation. Genetic and transgenic experiments show that they are functionally distinct, varying in *FLC* expression level and rate of epigenetic silencing. Allelic heterogeneity at this single locus accounts for a large proportion of natural variation in vernalization that contributes to adaptation of *A. thaliana*.

## Abstract (precis)

A key question in evolutionary biology is how molecular variation relates to phenotypic diversity. *Arabidopsis FLOWERING LOCUS C* (*FLC*) plays a key role in controlling vernalization—the acceleration of flowering by prolonged cold. Li et al. identify five functionally distinct *FLC* haplotypes, defined by noncoding sequence variation, which vary in *FLC* expression level and silencing. Allelic heterogeneity at this single locus accounts for a large proportion of variation in *Arabidopsis* vernalization. This study advances our understanding of adaptation and provides a new paradigm for analysis of complex traits.

Reproductive timing is central to plant adaptation through its major effect on fitness (Mitchell-Olds and Schmitt 2006). Many plants overwinter before flowering, thus aligning the transition to reproductive development with spring. In *Arabidopsis thaliana*, this process, called vernalization, involves expression and then subsequent epigenetic silencing of a floral repressor gene named *FLOWERING LOCUS C* (*FLC*) (Sheldon et al. 2000; Michaels 2009). *A. thaliana* accessions show extensive natural variation in vernalization (Nordborg and Bergelson 1999; Shindo et al. 2006; Alonso-Blanco and Mendez-Vigo 2014). Relatively rapid vernalization is thought to facilitate adaptation to habitats with high summer drought or other high mortality situations, whereas slow vernalization is thought to be an important adaptation to northern latitudes (Stinchcombe et al. 2004). In some cases, this variation has been mapped to the *FLC* locus itself (Coustham et al. 2012), but the extent to which *FLC* variation accounts for natural variation in vernalization response and the importance of this natural variation to adaptation remain important unanswered questions (Satake 2010). We exploited the extensive collection of genotyped *A. thaliana* accessions to characterize the genetic architecture of the *FLC* locus in worldwide accessions. Here, we describe the phenotypic variation conferred by the different *FLC* haplotypes, their geographical distribution, and the extent to which variation at *FLC* accounts for the phenotype. These data reveal that the extensive allelic heterogeneity at this single floral repressor gene can account for a major fraction of the natural variation in vernalization rate that underpins life history diversity in *A. thaliana.*

## Results and Discussion

**Figure 1.**
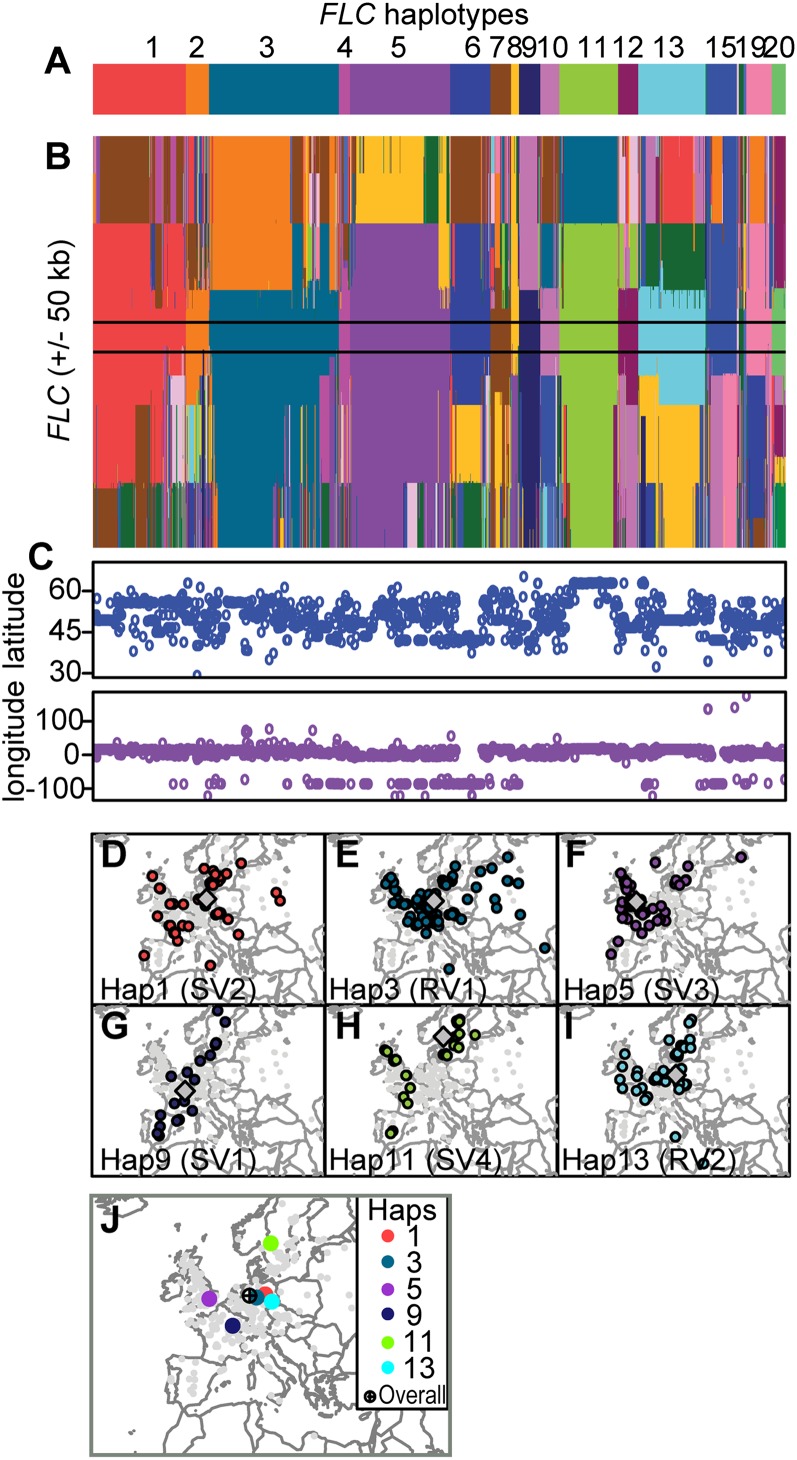
Different *FLC* haplotypes in 1307 worldwide *A. thaliana* accessions. (*A*) Color-coded groups are indicated with numbers. The rare Hap14 and Hap16–18 are not marked. (*B*) Haplotype structure of the ±50-kb *FLC* region in 1307 global *A. thaliana* accessions. Each accession is represented in a column, and each SNP in the *FLC* ±50-kb window is represented in a row. At each SNP position, colors indicate the most likely haplotype membership for each accession as determined by fastPHASE analysis. Accessions are ordered by haplotype at the *FLC* locus itself, which is delineated by solid black horizontal lines. The prominent vertically oriented color blocks indicate that haplotype sharing among accessions often extends beyond the *FLC* locus itself. (*C*) Latitude and longitude of the collection site corresponding to the accessions *above* (Horton et al. 2012). (*D*–*I*) Geographical distribution of collection sites of *A. thaliana* accessions carrying the five predominant and one intermediate frequency *FLC* haplotypes. (*J*) Centroid distribution of the *FLC* haplotypes.

Single-nucleotide polymorphism (SNP) data from the Regional Mapping panel project (Horton et al. 2012) identified 20 *FLC* haplotypes across the 1307 accessions (Fig. 1A), with five major haplotypes predominating (Hap1, Hap3, Hap5, Hap11, and Hap13), numbered according to sequence similarity. A Northern Swedish accession, Lov-1, that we studied previously fell into Hap9, which showed intermediate frequency (Coustham et al. 2012). In the worldwide samples, the *FLC* region is characterized by high population structure and extensive haplotype sharing (Fig. 1B). Hap1, Hap3, and Hap13 have broad geographical distributions with centroids in central Europe, while Hap5 is predominantly in the United Kingdom, and Hap9 and Hap11 tend to be near coasts (Fig. 1C–J).

**Figure 2.**
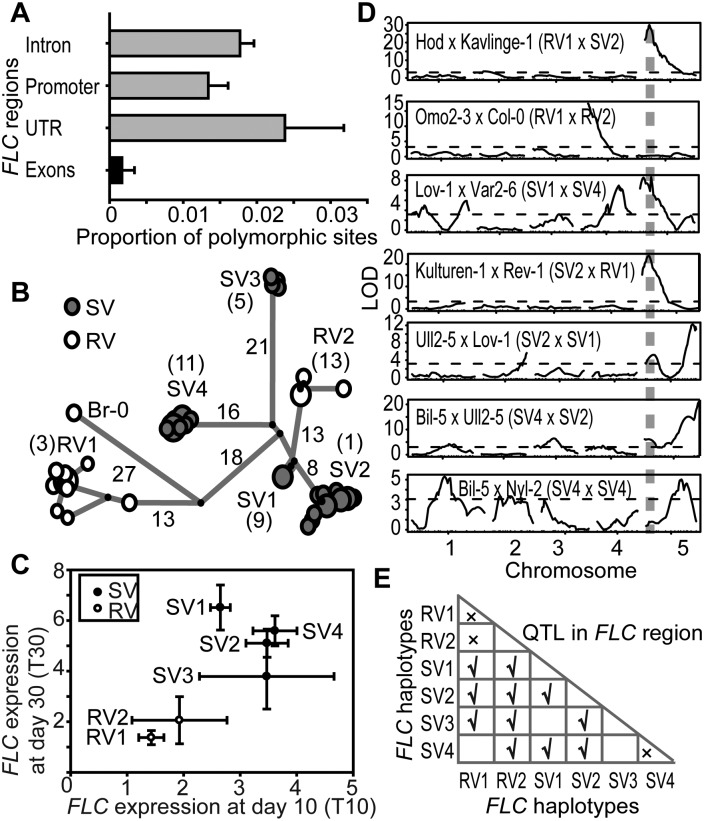
Functional differentiation of *FLC* haplotypes. (*A*) Distribution of genetic polymorphism (the mean [±SEM] proportion of polymorphic sites) across the 10-kb *FLC* region. (*B*) Haplotype analysis of 10-kb *FLC* genomic sequences from 47 *A. thaliana* accessions using FLUXUS network analysis. Circle size illustrates the frequency of the corresponding *FLC* haplotype. The number along the branch shows the number of nucleotide differences. Numbers in brackets indicate the corresponding haplotype numbers. (*C*) *FLC* expression of haplotypes containing *FLC* alleles with a slow vernalization (SV, black symbols) or rapid vernalization (RV, white symbols) response. Expression values shown are mean (±SEM, *n* = 2–13) from plants given 4 wk of cold followed by 10 d (T10) or 30 d (T30) of warm. Significant differences in vernalization response exist between haplotypes at 10 d (T10) and 30 d (T30) (Kruskal-Wallis test: H ≥ 18.42, d.f. = 5, *P* ≤ 0.002 in all tests). (*D*) QTL analysis on populations generated from crosses between accessions containing different *FLC* haplotypes. Dashed horizontal lines show the significance thresholds. The *FLC* region is indicated by the vertical dashed lines. (*E*) Summary of the presence of a QTL in the *FLC* region from *D* and Supplemental Table S3. The “✓” symbol indicates that there is a QTL in the *FLC* genomic region in the F2 population; the “×” indicates that there is no QTL.

We selected 47 *A. thaliana* accessions that represented the major haplotypes and generated a high-quality *FLC* sequence for each accession (Supplemental Table S1). Remarkably, the predicted amino acid sequences from these *FLC* alleles were identical, with very low polymorphism in the coding sequences but considerable nucleotide sequence polymorphism in noncoding regions (Fig. 2A,B). The functional significance of this noncoding polymorphism was assessed through analysis of the vernalization response of the 47 accessions, using a partial vernalization treatment to enhance phenotypic diversity. The vernalization response—degree of reactivation of *FLC* expression after 4 wk of cold—varied among accessions and grouped with haplotype (Fig. 2). Two haplotypes (Hap3 and Hap13) showed relatively rapid vernalization (RV) response and so were named RV1 and RV2. Four others (Hap9, Hap1, Hap5, and Hap11) showed a relatively slow vernalization (SV) response and so were named SV1–4 (Fig. 2B,C; Supplemental Fig. S1A,B). The reactivation of *FLC* expression after transfer back to the warm differed for each haplotype and correlated with flowering time (Supplemental Fig. S1C,D). Additionally, 114 Swedish *A. thaliana* accessions (Supplemental Table S2), which had been genotyped using the 250K SNP array (Horton et al. 2012), were also phenotyped for their vernalization response. Similar groupings of haplotypes with phenotypes emerged from this analysis (Supplemental Fig. S2A,B**)**. In general, therefore, accessions in the different *FLC* haplotype groups show distinct vernalization profiles that involve changes in silencing of *FLC* and altered flowering time.

The functional diversification of the different *FLC* haplotypes is supported by previous quantitative trait locus (QTL) analyses (Supplemental Table S3; Alonso-Blanco et al. 1998; Shindo et al. 2006; O’Neill et al. 2008; Li et al. 2010; Salome et al. 2011). Strong-effect QTL for vernalization response has been mapped over the *FLC* genomic region in populations generated from parents with different haplotypes: Col-0 (RV2) crossed to Lov-1 (SV1), Ull2–5 (SV2), or Var2–6 (SV4) (Shindo et al. 2006). To further test the functional diversity of *FLC* haplotypes, we undertook additional QTL analyses using F2 populations generated from crosses between accessions containing *FLC* alleles within and between haplotypes. In all crosses between the RV × SV accessions, a QTL was detected over the *FLC* genomic region, reinforcing the view that the haplotypes confer functionally distinct responses. No *FLC* QTL was detected in the cross between the RV1 × RV2 accessions due to their very similar vernalization phenotypes (Fig. 2D,E). This association between *FLC* alleles and flowering time was significantly reduced after 12 wk of vernalization compared with 4 wk, suggesting that the functional difference between haplotypes is cold exposure-dependent (Supplemental Fig. S2C,D).

**Figure 3.**
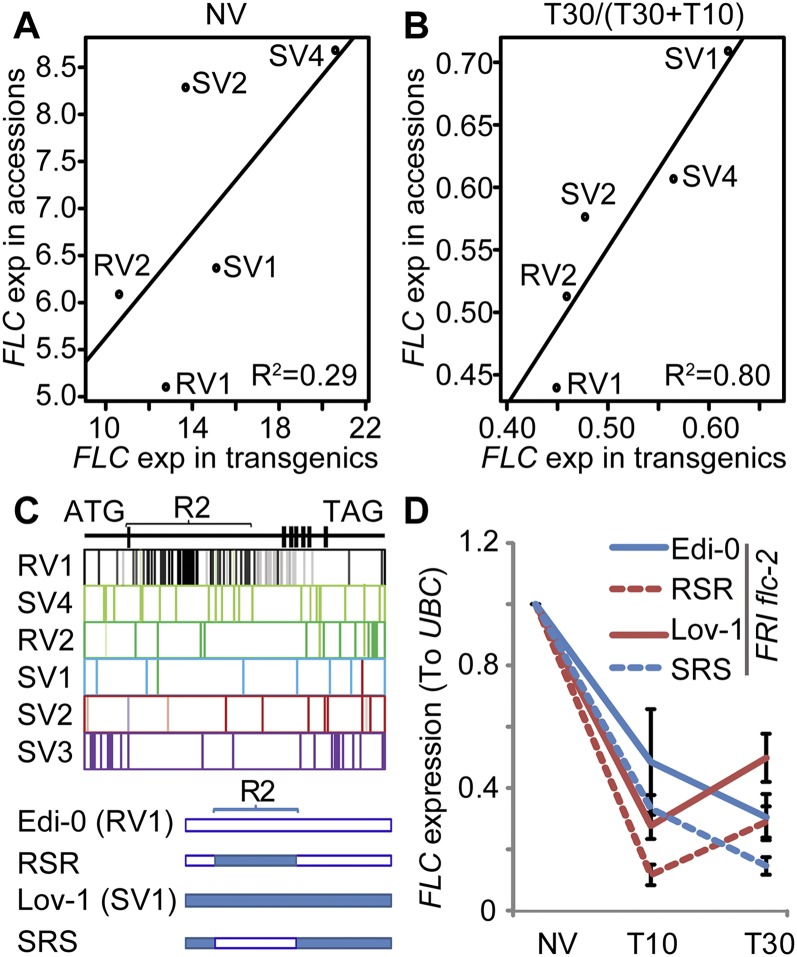
Transgenic analysis shows that noncoding *FLC* polymorphism plays a central role in vernalization response of natural *Arabidopsis* accessions. (*A*) Prevernalization *FLC* expression (nonvernalizaion [NV]) in transgenic lines correlates weakly with natural accessions. (*B*) Relative *FLC* expression [T30/(T30 + T10)] in transgenic lines correlates significantly with expression in natural accessions. R^2^ shows the explained amount of variation; the corresponding *P*-values are 0.201 and 0.025 in *A* and *B*. (*C*) Schematic illustration of *FLC* polymorphisms and the constructs of R2 chimeras. The SNPs defining the different *FLC* haplotypes are shown by the vertical bars. The bar transparency indicates the diversity of each SNP within each haplotype. The bars shared between haplotypes signify shared SNPs. The *FLC* gene structure is shown *above*: Filled black squares are exons, and horizontal lines are introns and untranslated regions. RSR and SRS chimeras show the exchange of R2 regions between Edi-0 and Lov-1 *FLC* alleles. (*D*) *FLC* expression analysis of transgenic *FRI flc-2* plants containing chimeric *FLC* alleles. Plants were given either no cold (NV) or 4 wk of cold followed by 10 d (T10) or 30 d (T30) of warm. Values are means ±standard error from three biological repeats.

To conclusively demonstrate that *FLC* variation is the major contributor to the QTL, we generated transgenic lines differing only in their *FLC* allele. Twelve-kilobase *FLC* genomic clones from the *A. thaliana* accessions Edi-0, Col-0, Lov-1, Ull2-5, and Var2-6, representing haplotypes RV1, RV2, SV1, SV2, and SV4, respectively, were transformed individually into *FRI flc-2* (Michaels and Amasino 2001). *FLC* expression in the transgenic lines was compared with that from the natural accessions both with and without vernalization. The differential response to vernalization shown by the *FLC* haplotypes was recapitulated in the transgenic plants (R^2^ = 0.80), whereas the correlation of prevernalization *FLC* expression between accessions and transgenic lines was weaker (R^2^ = 0.29) (Fig. 3A,B). The transgenic analysis therefore shows that the SNPs defining the *FLC* alleles confer the vernalization response characteristic of the parent *A. thaliana* accession, with *trans*-regulation playing a greater role in prevernalization *FLC* expression. These data enable us to conclude that noncoding *cis* variation within *FLC* is the major factor determining differential vernalization response in the characterized *A. thaliana* population.

Analysis of chimeric *FLC* fusions previously showed that *cis* polymorphism quantitatively modulates a chromatin silencing mechanism underpinning the epigenetic regulation of *FLC* in one Northern Swedish accession (Coustham et al. 2012). To help define which of the many nucleotide differences between haplotypes determined variation in vernalization response, we first looked for natural recombination events across *FLC* genomic regions in accessions (Martin et al. 2010) and identified one that we called R2 (Supplemental Fig. S3A). Polymorphism in the R2 region showed the strongest association with the maintenance of *FLC* silencing after vernalization (Supplemental Fig. S3B). This region includes the nucleation region important for *FLC* silencing; the VRE (vernalization response element), defined as important for maintenance of silencing (Sheldon et al. 2002; Finnegan and Dennis 2007; Angel et al. 2011); and a large number of the SNPs defining the haplotype RV1 (Fig. 3C). We isolated and swapped the *FLC* R2 region reciprocally between Lov-1 (SV1) and Edi-0 (RV1) *FLC* alleles, which show slow and rapid silencing, respectively (Fig. 3C). The vernalization response of multiple transgenic lines was then compared. The SV1/RV1/SV1 (SRS) chimeric *FLC* shows stable *FLC* silencing after 4 wk of cold, which results in a rapid vernalization response compared with its SV1 *FLC* parent (Fig. 3D). Thus, the SRS vernalization response matches that of the RV1 accession rather than the SV1 accession. Similarly, the RV1/SV1/RV1 (RSR) chimera shows a vernalization response similar to the SV1 accession despite containing only a small part of the SV1 *FLC* allele (Fig. 3D). These results demonstrate that polymorphisms within R2 from RV1 and SV1 contribute to the differences in *FLC* silencing after 4 wk of vernalization, respectively. This region also contains the four single-nucleotide changes that cause the differential silencing of Lov-1 (SV1) and Col-0 (RV2) *FLC* alleles (Coustham et al. 2012). Remarkably, the Edi-0 (RV1) and Col-0 (RV2) *FLC* alleles do not share any polymorphisms that are not also found in other *FLC* haplotypes (Figs. 2B, 3C), demonstrating that the causative nucleotide changes that distinguish slow and rapid vernalization have evolved multiple times. The recombination event characterizing the RV1 haplotypes, the relative lack of variability of SNPs in the functional R2 region, and their relatively broad geographical distribution (Hap3/RV1) (Fig. 1E) suggest a relatively recent spread of RV1 accessions, potentially indicating a strong advantage of rapid vernalization in some locations.

**Figure 4.**
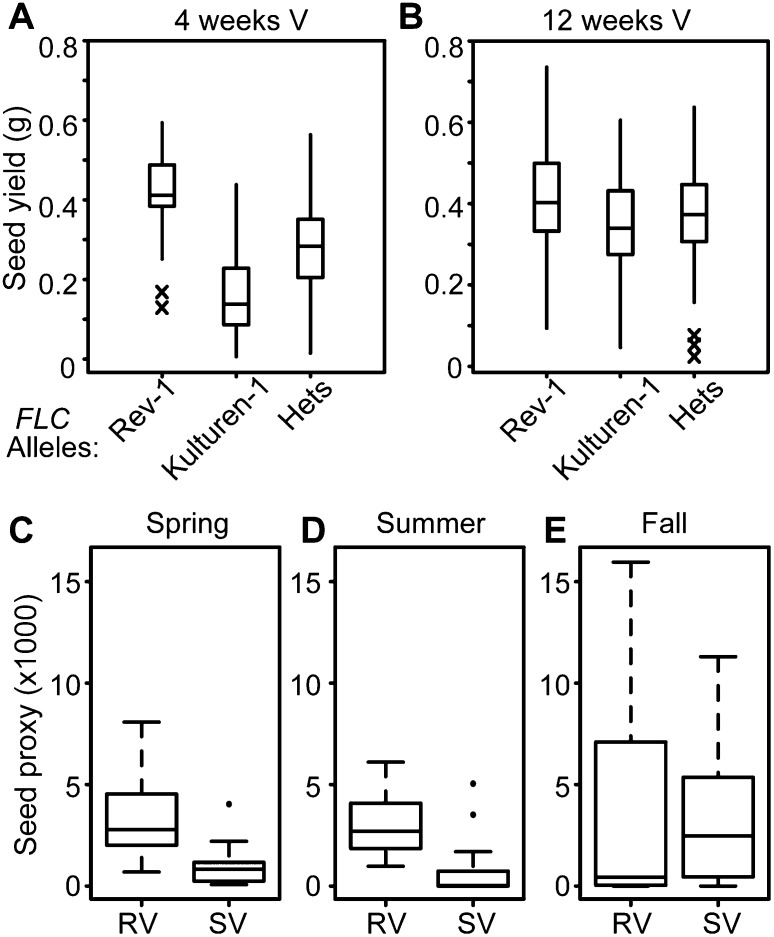
Comparison of total seed production between plants with different vernalization responses. (*A*,*B*) Comparison of seed yield from F2 plants homozygous or heterozygous for the different *FLC* alleles after 4 wk (*A*; ANOVA, R^2^ = 0.41, F_2227_ = 80.43, *P* < 0.001) and 12 wk (*B*; ANOVA, R^2^ = 0.028, F_2276_ = 4.97, *P* = 0.008) of vernalization. Crosses indicate statistical outliers that fall more than three standard errors away from the mean. (*C*–*E*) Seed proxy values as a measure of fitness of field-grown accessions. The data set included only accessions with active *FRIGIDA* alleles (16 accessions from haplotypes RV1 and RV2 and 23 accessions from haplotype SV1-4). *FLC* haplotypes with rapid and slower vernalization response phenotypes were analyzed. Differences in yield were measured in field experiments in Norwich as in Fournier-Level et al. (2011). *P*-values are calculated from population structure-corrected ANOVA (see the Materials and Methods) (*C*; *P* = 0.0023), summer sowing (*D*; *P* = 0.00017), and fall sowing (*E*; *P* = 0.99).

Consistent with this, faster vernalization has been predicted to confer a selective advantage to plants where avoidance of summer drought or other high risk is beneficial (Caicedo et al. 2004; Stinchcombe et al. 2004, 2005; Scarcelli et al. 2007; Satake 2010). We analyzed differences in fitness-related traits conferred by different *FLC* alleles in F2 populations (Fig. 4; Supplemental Fig. S4). After a relatively short vernalization (4 wk), the slow vernalization (Kulturen-1) *FLC* allele associated with lower seed yield than the rapid vernalization (Rev-1) *FLC* allele. After longer vernalization (12 wk), both alleles produced a similar seed yield (Fig. 4A,B; Supplemental Fig. S4A). Total seed weight represented an increased production of seed number, as no significant difference was found in the weight of each seed (Supplemental Fig. S4B). High fecundity has been associated with later flowering (Rédei 1962); however, for these rapid vernalization and slow vernalization alleles, an inverse relationship was found between flowering time and seed production after 4 wk of vernalization (Supplemental Fig. S4C). A similar relationship between vernalization response and fitness was also found for other rapid vernalization/slow vernalization allele combinations (Supplemental Fig.S4D). These data suggest that in some conditions, particularly after short cold periods, faster vernalization response can lead to enhanced fitness. To further explore the association of *FLC* haplotypes with fitness, we analyzed the field data of *A. thaliana* accessions grown under different natural seasonal conditions in Norwich (United Kingdom), where spring-, summer-, and fall-germinating cohorts are observed (Fournier-Level et al. 2011). Accessions with slow vernalization haplotypes produced less seed compared with rapid vernalization accessions in spring and summer sowings (which experienced only brief exposure to vernalizing temperatures), but fitness was similar between all groups in fall sowings (with exposure to extended cold such that all plants were fully vernalized) (Fig. 4C–E). These data encourage further extensive transplantation experiments that thoroughly investigate fitness differences between the haplotypes.

The emerging picture is that functional differences in vernalization have evolved predominantly through *cis* polymorphism at *FLC.* Multiple alleles have arisen and been maintained in the *A. thaliana* population due to their contributions to life history diversity. Use of *FLC* genomic sequence from a close relative, *Arabidopsis lyrata*, did not help define which of the haplotypes are ancestral because of the high sequence divergence and large number of indels in the functionally important intronic regions. However, rapid vernalization might have arisen through selection pressure on the length of the plant life cycle due to high mortality from drought (Hoffmann 2005; Satake 2010), herbivory (Parachnowitsch and Lajeunesse 2012), pathogens (Kemen et al. 2011), agriculture, or human disturbance. The broader climate envelope of *A. thaliana* compared with its close relatives, which extends beyond the cool temperate regions into more Mediterranean climates, argues that faster vernalization could have increased the *A. thaliana* range (Hoffmann 2005). This and related studies should provide important knowledge needed to assess plant response to climate change.

## Materials and methods

### Plant materials and growth condition

Transgenic lines were obtained as described previously (Coustham et al. 2012). Plants were vernalized for 4 or 12 wk at 4°C and harvested for *FLC* expression analysis 10 d (T10) and 30 d (T30) after transfer from cold, along with nonvernalized plants (NV). Flowering time was assayed as days after bolting when stems reached 3 cm in height.

### Plant fitness estimation

Controlled environment conditions were as follows: Plants were vernalized for 4 or 12 wk at 4°C under short-day conditions (8 h light) and moved to controlled environment conditions at 22°C under a long-day photoperiod (16 h light). Soil was from The Scotts Company Ltd., Sphagnum Moss peat (pH 5.5–6.0), with fertilizer added (milligram/liter): N150, P200, and K200. Twenty-five percent grit was added before use. Pot size was 5 × 5 cm. Plants were grown individually in each pot. In the greenhouse, total seed was collected from each plant after siliques had matured and dried naturally and was weighed.

In the field, plants of 39 accessions were grown in common gardens at the John Innes Center in spring, summer, and fall plantings as described previously (Fournier-Level et al. 2011). Fitness was estimated as the product of silique number at senescence and length of a representative silique (a proxy for seed number) for each individual (Fournier-Level et al. 2011).

### FLC haplotype analysis using FLUXUS network analysis

*FLC* genome sequences were aligned using ClustalW with manual corrections in Mega5 (Tamura et al. 2011) and analyzed in FLUXUS network software using median joining setting (Bandelt et al. 1999).

### FLC expression analysis

*FLC* expression data were obtained as described previously (Box et al. 2011). We generated 50 independent transgenic lines for each construct using the *Agrobacterium tumefaciens* floral dip technique. To overcome the variability in expression between transgenic lines, we pooled a random 10 seedlings from each of these 50 lines into a single sample. RNA was extracted from this pool and analyzed for *FLC* expression using quantitative RT–PCR as described in Box et al. (2011). For each time point, three independent pools of the 50 transgenic line pools were assayed.

### Box plot and statistical analysis

Box plot and statistical analysis of flowering time and seed production were carried out in the GENESTAT package with the default setting (Ripatti et al. 2009).

### Genotyping and QTL analysis of F2 populations

F2 populations were genotyped using a 384-SNP Illumina GoldenGate genotyping assay (http://www.illumina.com/technology/goldengate_genotyping_assay.ilmn). Genome Studio software (http://www.illumina.com/software/genomestudio_software.ilmn) and custom-made scripts in R software (http://www.R-project.org) were used for SNP calling.

Quantitative trait loci mapping was performed on raw phenotypic values without any transformation. QTL analyses were carried out using the default parameters of the scanone function implemented in the R/qtl package (Broman et al. 2003). The LOD significance threshold was determined by permutation test (1000 permutations, significance level of 5%) for each F2 population.

### M&M test of the correlation between FLC polymorphism and gene expression

A general linear model (GLM) with T30/(T30 + T10) as response variable and SNP as the predictor was analyzed for each SNP separately. The F coefficient (adjusted MS/error variance) was calculated and plotted against the SNP position. The strength of the association of each SNP and the response variable was expressed by calculating the effect size estimator *η*^2^. This statistic expresses the ratio of variance explained in the dependent variable by a SNP. This analysis shows that SNPs in the R2 region (Supplemental Fig. S5B) explain ∼22.1% of the variation inT30/(T10 +T30) (*η*^2^ = 0.221), which is considered a large effect size.

### fastPHASE haplotype analysis

SNPs in a window ±50 kb around *FLC* were extracted from a preimputation version of the Regional Mapping Project SNP panel described in Horton et al. (2012). These SNPs were used as the input into fastPHASE version 1.4.0 (Scheet and Stephens 2006), which was run using the default settings, with the exception of invoking the −Pzp option to output raw likelihood values. For each SNP in the analysis, the cluster membership with the highest likelihood was designated as the haplotype for that SNP.

### Population structure-corrected ANOVAs for seed proxy analysis in accessions

A standard approach for accounting for such genomic background effects is linear mixed models (Vilhjálmsson and Nordborg 2013). We assessed the significance of phenotypic differences between haplotypes by carrying out an ANOVA on the residuals after subtracting the best linear unbiased predictors (BLUPs) of the effect explained by the genomic background. The BLUPs were estimated using EMMAX (Kang et al. 2010) with a kinship matrix estimated from the Regional Mapping Project data (Horton et al. 2012).
